# Supplementary material for: Patients with Thyroid Dyshormonogenesis and DUOX2 Variants: Molecular and Clinical Description and Genotype–Phenotype Correlation
Source: Int J Mol Sci. 2024 Aug 3;25(15):8473. doi: 10.3390/ijms25158473 (PMC11313534; doi:10.3390/ijms25158473)
Supplement: Supplementary file 1 [file ijms-25-08473-s001.zip › ijms-3116823-supplementary.pdf]

Patients with thyroid dyshormonogenesis and *DUOX2* variants: molecular and clinical description and genotype-phenotype correlation

Noelia Baz-Redón, María Antolín, María Clemente, Ariadna Campos, Eduard Mogas, Mónica Fernández-Cancio, Elisenda Zafon, Elena García-Arumí, Laura Soler, Núria González, Cristina Aguilar, Núria Camats-Tarruella, Diego Yeste

<u>SUPPLEMENTARY MATERIALS</u>

Table S1: Clinical manifestations and genetic results of excluded patients with *DUOX2* gene variants that could not explained their thyroid dyshormonogenesis phenotypes.

#Patient	TSH neonatal screening (mIU/L)	TSH conf (mIU/L)	FT4 conf (ng/dL)	Tg conf (ng/mL)	Scintigraphy/ Ultrasonography	Perchlorate discharge test	Final CH diagnosis	Nucleotide change (NM_014080.4)	AA change (NP_054799.4)	Pathogenicity (ACMG)	Zygosity	Familial segregation	Variants in other genes
**TDH-2**	126.7	67.7	NA	NA	normocaptant /normal	0% (negative)	Permanent[2]	c.514-49G>A		LB	Het	Mo	
**TDH-4**	125	75	1	NA	normocaptant /NA	6% (negative)	Severe permanent[1]	c.1498T>C	p.(Phe500Leu)	VUS	Het	Fa	*TG* [c.7851C>G; p.(Tyr2617Ter)+ c.8144G>C; p.(Cys2715Ser)]
**TDH-15**	27.5	83.1	0.94	1073	hypercaptant/ NA	NA	NR no follow-up	c.3391G>A	p.(Ala1131Thr)	VUS	Het	NA	*TG* [c.416G>A; p.(Trp139Ter)]
**TDH-19**	39.9	54.5	1.6	30.8	hypocaptant/ NA	NA	Transient[1]	c.943+11G>C		LB	Het	NA	
**TDH-20**	83.7	64.2	1.40	121	hypocaptant/ NA	NA	Transient[2]	c.3114C>T/ c.3694-24T>G	p.(Tyr1038=)/-	LB/LB	NA	NA	
**TDH-21***	18.6	90.2	1.9	41.1	hypocaptant/ NA	NA	Transient[2]	c.3041C>T	p.(Ala1014Val)	LB	Het	NA	*TG* [c.1267C>T; p.(Arg423Cys)]
**TDH-31***	56.1	63.8	1.52	49.3	normocaptant /NA	NA	Transient[1]	c.3041C>T	p.(Ala1014Val)	LB	Het	NA	

*: siblings; conf: diagnostic confirmation values; TSH: thyroid-stimulating hormone; FT4: free thyroxine; Tg: thyroglobulin; ref: reference values; CH: congenital hypothyroidism; AA: amino acid; NA: non available; NR: non reevaluated; VUS: variant of uncertain significance; LB: likely benign; Het: heterozygous; Mo: carrier mother; Fa: carrier father. [1]Final diagnosis classification according to TSH and FT4 levels at reevaluation (TSH<5mIU/L transient hypothyroidism; TSH 5-10mIU/L hyperthyrotropinemia; TSH>10mIU/L, FT4 normal mild permanent hypothyroidism; TSH>10mIU/L, FT4<0.8ng/dL severe permanent hypothyroidism). [2]Classification of congenital hypothyroidism severity according to the need for levothyroxine treatment.

Table S2: Clinical characteristics and anthropometric neonatal parameters of patients with confirmed thyroid dyshormonogenesis diagnosis by variants in the *DUOX2* gene.

#Patient	Gender	Age	Gestational age (weeks)	Birth weight (g)	Birth weight (SD)	Birth lenght (cm)	Birth lenght (SD)
**TDH-1**	M	24	41	NA	NA	NA	NA
**TDH-3**	F	17	39	3600	1.1	51.0	0.9
**TDH-5**	M	15	40	4070	1.7	54.0	2.1
**TDH-6**	M	13	37	NA	NA	NA	NA
**TDH-7**	F	13	42	3170	-0.8	50.0	-0.3
**TDH-8**	M	13	35	1770	-1.9	42.0	-2.19
**TDH-9**	F	11	40	3260	-0.05	49.0	-0.48
**TDH-10**	F	9	38	3300	0.84	49.0	0.19
**TDH-11**	M	8	36	2875	0.6	47.0	-0.2
**TDH-12**	F	8	37	2430	-0.9	51.0	1.5
**TDH-13**	F	8	41	3110	-0.6	48.0	-1.3
**TDH-14**	F	8	40	4120	2.2	53.0	1.9
**TDH-16**	F	7	41	2985	-0.9	48.0	-1.3
**TDH-17**	F	6	38	NA	NA	NA	NA
**TDH-18**	M	6	41	3520	0.1	52.5	1.0
**TDH-22**	F	5	41	3250	-0.25	50.0	-0.06
**TDH-23**	M	5	39	3300	0	48.0	-1.18
**TDH-24**	M	5	39	3225	-0.19	NA	NA
**TDH-25**	M	4	38	3600	1.11	51.5	1.2
**TDH-26**	F	3	39	2880	-0.82	47.5	-1.16
**TDH-27**	F	3	33	1620	-0.94	43	0
**TDH-28**	F	3	29	830	-1.5	NA	NA
**TDH-29**	M	2	40	3600	0.51	49	-0.83
**TDH-30**	M	2	37	2265	-1,44	44.5	-2,1
**TDH-32**	M	2	37	3000	0.21	48	-0.26
**TDH-33**	F	2	41	NA	NA	NA	NA
**TDH-34**	M	2	39	3550	0.62	49.5	-0.29
**TDH-35**	M	1	40	4000	1.5	NA	NA
**TDH-36**	F	1	39	3710	1.41	51	0.94
**TDH-37**	F	1	NA	NA	NA	NA	NA
**TDH-38**	M	16	NA	NA	NA	NA	NA

F: female; M: male; SD: standard deviation; NA: non available data.
